# Deposition of calcium in an *in vitro* model of human breast tumour calcification reveals functional role for ALP activity, altered expression of osteogenic genes and dysregulation of the TRPM7 ion channel

**DOI:** 10.1038/s41598-018-36496-9

**Published:** 2019-01-24

**Authors:** Shane O’Grady, Maria P. Morgan

**Affiliations:** Molecular and Cellular Therapeutics, Royal College of Surgeons in Ireland, 123 St. Stephen’s Green, Dublin, 2 Ireland

## Abstract

Microcalcifications are vital mammographic indicators contributing to the early detection of up to 50% of non-palpable tumours and may also be valuable as prognostic markers. However, the precise mechanism by which they form remains incompletely understood. Following development of an *in vitro* model using human breast cancer cells lines cultured with a combination of mineralisation-promoting reagents, analysis of calcium deposition, alkaline phosphatase (ALP) activity and changes in expression of key genes was used to monitor the calcification process. Two cell lines were identified as successfully mineralising *in vitro*, MDA-MB-231 and SKBR3. Mineralising cell lines displayed higher levels of ALP activity that was further increased by addition of mineralisation promoting media. qPCR analysis revealed changes in expression of both pro- (RUNX2) and anti- (MGP, ENPP1) mineralisation genes. Mineralisation was suppressed by chelation of intracellular Ca^2+^ and inhibition of TRPM7, demonstrating a functional role for the channel in formation of microcalcifications. Increased Mg^2+^ was also found to effectively reduce calcium deposition. These results expand the number of human breast cancer cell lines with a demonstrated *in vitro* mineralisation capability, provide further evidence for the role of an active, cellular process of microcalcification formation and demonstrate for the first time a role for TRPM7 mediated Ca^2+^ transport.

## Introduction

Breast cancer survival rates have risen significantly in recent decades, due to a combination of improved treatments options and early detection. Although some controversy exists within the literature^1,2^, the majority of studies have shown the adoption of mammography programmes to confer a significant decrease in breast cancer mortality^3,4^. Breast cancer can be detected by a number of mammographic features including density, architectural distortions and the presence of microcalcifications. The clinical relevance of microcalcifications was first identified in 1951 by Leborgne, who recognised they could constitute the sole mammographic indicator of carcinoma^5^. Mammographic detection of microcalcifications has since come to be regarded as a highly useful marker of breast cancer, with somewhere between 30 and 50% of non-palpable tumours found in screening identified solely due to the presence of microcalcifications^6,7^. They are also present in the majority of ductal carcinoma *in situ* (DCIS) cases^8^. Microcalcifications detected by mammography can be categorized based on their size, shape, chemical composition and spatial distribution within the breast, allowing for assessment as a benign or suspicious finding^9^. In addition to their utility as a detection marker, the presence of microcalcifications within a breast tumour may also be of prognostic significance, with many studies highlighting links between calcifications and poor prognosis^10,11^, high tumour grade^12,13^ and increased risk of recurrence^11,14^. Microcalcifications also display a significant association with human epidermal growth factor receptor 2 (HER2) overexpression^14,15^ although their relationship with hormone receptor (estrogen or progesterone) status is unclear as various studies have found both positive^13,16,17^ and negative associations^11,18,19^ or no association at all^20,21^.

Despite their significant contribution to the detection of breast tumours, the detailed mechanism by which microcalcifications form remains unknown. Previous research from our lab established the first *in vitro* model of microcalcification formation^22^. Culturing the murine 4T1 cell line with similar pro-mineralisation reagents as studies on physiological osteoblast mineralisation resulted in deposition of hydroxyapatite, a form of calcification commonly associated with malignancy^22,23^. This work demonstrated for the first time the role of an active, cell-regulated process of mammary mineralisation. Since development of our model of breast microcalcification formation, other groups have utilised it to investigate novel players in the process, including the secretory pathway Ca^2+^-ATPases transporters^24^ and carbonic anhydrase^25^.

Formation of microcalcifications has been hypothesised to result from a dysregulation of calcium transport pathways in conjunction with ectopic expression of bone-associated genes^23,26,27^. This is supported by findings of altered expression levels in several bone-associated proteins in breast tumours with associated calcifications^28–31^. However, the role of calcium transport proteins remains unclear. In addition, the majority of our prior work utilised the murine 4T1 cell line and although human breast cancer cell lines were also assessed, the number of cell lines examined was low and not representative of the diversity of breast cancer subtypes. It is not currently known if any particular subtypes of human breast cancer cell lines are more likely to mineralise under *in vitro* conditions. A thorough examination of the mineralisation potential of a representative selection of human breast cancer cells is thus merited.

## Materials and Methods

### Cell lines and media

MDA-MB-231, SKBR3 and MCF7 cells were originally sourced from ATCC. HER2 overexpressing MCF7 cells^32^ (MCF7-HER2) were a kind gift from Professor Dennis Slamon, (University College Los Angeles, USA) and Dr Norma O’Donovan, (Dublin City University, Ireland). All cell lines were grown in DMEM media supplemented with 10% foetal bovine serum and 1% penicillin/ streptomycin. Media used for MCF7 cells also contained 0.01 mg/ml bovine insulin. All cell culture reagents were purchased from Labtech International (East Sussex, U.K.) and Sigma-Aldrich (Arklow, Ireland).

### Assessment of mineralisation

Cells to be tested for mineralisation capability were seeded into 6 well tissue culture plates in regular growth media and grown to 70–80% confluence. Media was removed and either replaced with fresh growth media (Control), media supplemented with an osteogenic cocktail (OC; 10 mM β-glycerophosphate and 50 µg/mL ascorbic acid) or media supplemented with OC and 100 nM dexamethasone (Dex). In some experiments, exogenous bovine alkaline phosphatase (ALP) (Sigma) was included in culture media at a concentration of 1 U/mL. The ALP inhibitor levamisole (Santa Cruz) was utilised at a concentration of 100 µM. Cells were grown under these conditions for up to 28 days, with half the media in each well changed twice a week. Mineralisation was assessed on a weekly basis, using Alizarin Red S and von Kossa staining, as well as the o-cresolphthalein complexone (OCP) calcium assay.

### Histological staining

Wells to be stained were fixed in a 10% formaldehyde-PBS solution for 30 minutes. Alizarin Red S staining was performed by incubation for 4 minutes with Alizarin Red S solution (2%, pH 4.4). The staining solution was subsequently removed and each well was washed 4 times with distilled water to reduce nonspecific binding. Wells were allowed to air dry then analysed by light microscopy. Bright red staining indicates a positive result for calcium.

von Kossa staining was carried out on separate wells, to verify Alizarin results. A 5% silver nitrate solution was applied for 1 hour under a bright light. This was followed by a 2 minute incubation with 5% sodium thiosulphate. After removal of the sodium thiosulphate and 3 washes with distilled water, plates were allowed to air dry before examination by light microscopy. Dark brown/black staining indicates a positive result for calcium phosphate.

### Alkaline phosphatase assay

To measure alkaline phosphatase (ALP) activity, cells were washed once with cold PBS, scraped into 1 mL PBS, pelleted by 5 minutes centrifugation at 2,000 RPM and resuspended in 100 µL ALP buffer (100 mM Tris-HCL pH 9.5, 100 mM NaCl, 5 mM MgCl, 1% Triton X-100). For analysis, ALP samples were diluted with ALP buffer (typically a 1:3 to 1:50 dilution). 50 µL of each sample was added to a flat bottom, clear plastic 96-well plate along with 50 µL of p-nitrophenyl phosphate (pNPP) chromogenic substrate (Sigma). A standard curve of purified bovine ALP (Sigma) was prepared in the range of 0–100 mU/mL using the batch information sheet to calculate units of enzymatic activity. Samples were incubated at 37 °C for 30 minutes and analysed by absorbance at 405 nm. All results were normalised to protein content as determined by BCA assay (Sigma).

### Quantitative o-cresolphthalein complexone (OCP) calcium assay

Samples for the OCP assay were generated by removal of medium from each well, rinsing once with PBS and incubating with 500 µL of 1 M nitric acid for 1 hour to dissolve any calcium deposits. Samples were diluted in 1 M nitric acid to within range of the assays standard curve (typically a 1:2–1:100 dilution). 70 µL of each dilution was added to a 96 well plate in triplicate followed by 70 µL OCP solution (Sigma) and 175 µL of amino-2-methyl-1-propanol (AMP; Sigma). A standard curve was generated by diluting a Ca^2+^ solution (Sigma) in nitric acid to a range of 0–10 ppm. Absorbance was read at 572 nm on a Wallac plate reader.

### Quantitative real-time PCR

Total RNA was extracted using Trizol (Thermo Fisher Scientific, Dublin, Ireland) following standard protocol. 1 µg RNA was used to generate cDNA with the High-Capacity cDNA Reverse Transcription Kit (Thermo Fisher Scientific) following manufacturers recommendations. Changes in gene expression were analysed using the SensiFAST SYBR Lo-ROX qPCR kit (Bioline, London, UK) in an AB7500 qPCR system using the following temperature profile: 95 °C for 2 minutes followed by 40 cycles of 95 °C for 15 seconds (denaturation), 60 °C for 15 seconds (annealing) and 72 °C for 30 seconds (elongation). Results were normalised to a housekeeping gene (18S) and analysed using the delta-delta-Ct (ddCt) method. Primer sequences (Sigma) are listed below (Table 1).

**Table 1:** Primer sequences utilised for qPCR analysis.

Target	Forward primer	Reverse primer
18 S	AACCCGTTGAACCCCATT	CCATCCAATCGGTAGTAGCG
ENPP1	CAAAGGTCGCTGTTTCGAGAG	TGCACGTCTCCTGGTAATCTAAA
MGP	TCCGAGAACGCTCTAAGCCT	GCAAAGTCTGTAGTCATCACAGG
RUNX2	CCTGAACTCTGCACCAAGTC	GAGGTGGCAGTGTCATCATC
TRPM7	CAGAAACCAAGCGCTTTCCT	AATTCAACGGCCAACTGACC

### siRNA knockdown

siRNA knockdown was performed using Lipofectamine RNAiMAX transfection reagent (Thermo Fisher Scientific) and Sigma pre-designed siRNA (Negative-control; SIC001, TRPM7; SASI_Hs01_00120022). Transfection was performed on actively dividing cells at approximately 70–80% confluency. Briefly, 250 µL serum free OptiMEM media was added to two separate Eppendorph tubes. 6 µL Lipofectamine was added to one tube, and 2 µL of 10 µM siRNA was added to the second tube. The two tubes were then combined and mixed by gentle pipetting before incubation at room temperature for 15–20 minutes. Finally, 500 µL of the transfection mix was added dropwise to cells in a 6 well plate, containing 1.5 mL DMEM. Plates were gently rocked to ensure even distribution of transfection complexes, and returned to the incubator. Changes in gene expression was assessed 72 hours post transfection by qPCR.

### Statistical analysis

All statistical analysis was carried out using GraphPad Prism 7 software. Student t-tests were used to compare two treatment groups. Differences between multiple treatment groups were analysed by one-way or two-way ANOVA, with post-hoc analysis to confirm significance. A p-value of less than 0.05 was deemed statistically significant.

## Results

### *In vitro* mineralisation capability of a panel of human breast cancer cell lines

Previous work from our group demonstrated successful mineralisation in breast cancer cell lines grown in media supplemented with an osteogenic cocktail (OC), consisting of β-glycerophosphate and ascorbic acid. To identify the specific mineralisation requirements for cell lines, OC was used both with and without dexamethasone (Dex), which has previously been shown to induce activation of pro-mineralisation signalling pathways^33^ in a cell line specific manner^22,23^.

**Figure 1:**
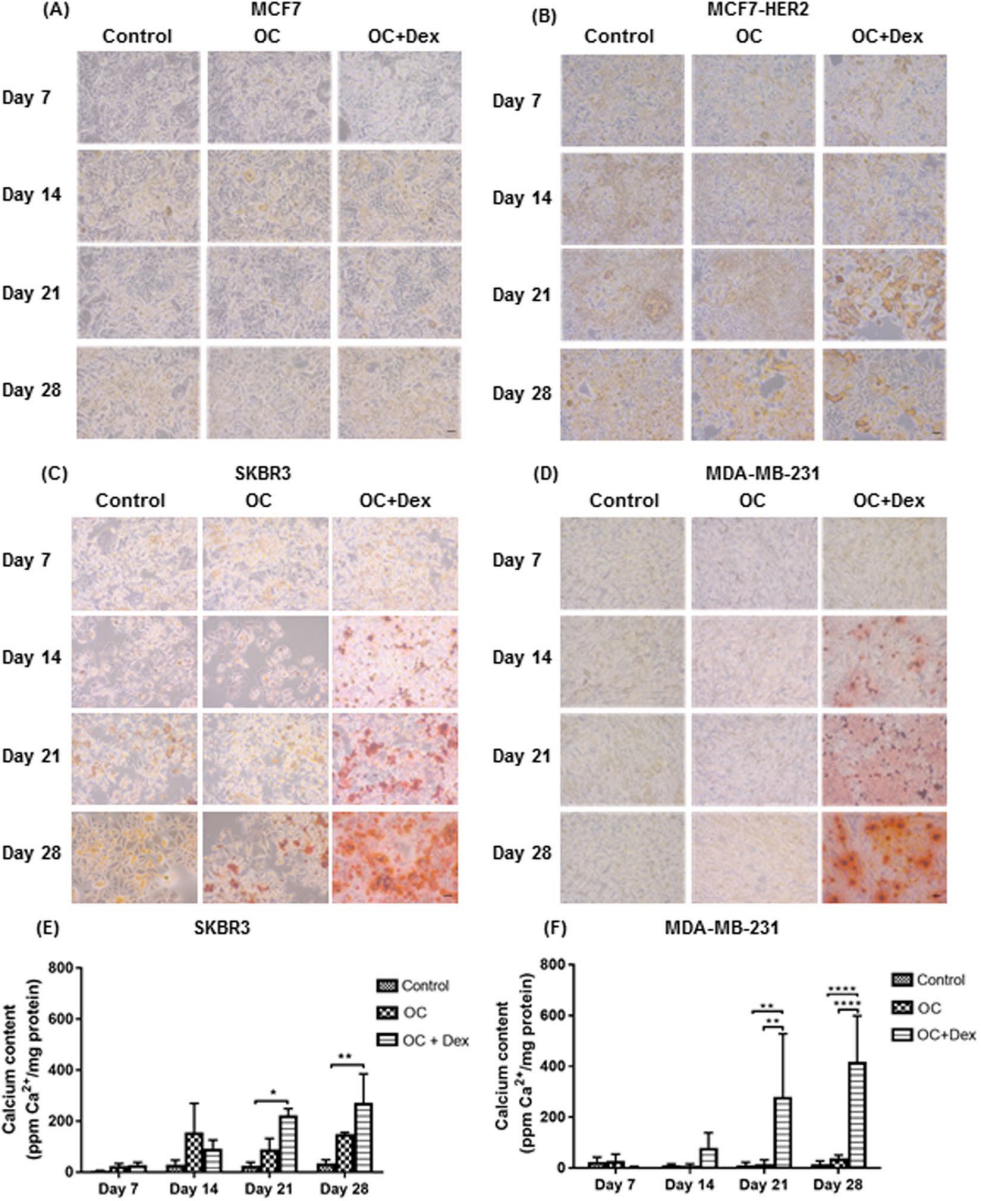
Establishment of a human *in vitro* model of microcalcification formation. Representative Alizarin Red S staining of MCF7 (**A**), MCF7-HER2 (**B**), SKBR3 (**C**) and MDA-MB-231 (**D**) cells over time, shown at 10X magnification (n = 3). Control (regular growth media), OC (osteogenic cocktail) (50 µg/ml ascorbic acid and 10 mM β-glycerophosphate) Dex (100 nM dexamethasone). Scale bar represents 50 µm. Calcium content of SKBR3 (**E**) and MDA-MB-231 (**F**) cells as determined by o-cresolphthalein calcium assay and normalised to protein. Each point represents the mean + SD (n = 3), two-way ANOVA. *p < 0.05, **p < 0.01, ****p < 0.0001.

We first aimed to extend the number of human breast cancer cell lines with a characterised mineralisation capability. The luminal MCF7 cell line was examined first, by culturing for 28 days in control media, or media supplemented with OC or OC with 100 nM dexamethasone (OC + Dex). Mineralisation, as examined via Alizarin Red S staining was negative at all tested time points, indicating no mineralisation capability in this cell line under tested conditions (Fig. 1A). Cells were also stained using the von Kossa protocol, which produced similarly negative results (data not shown). Several studies have found increased HER2 expression in patients presenting with microcalcification associated breast tumours^15,34,35^. However, a potential role between the HER2 receptor and the *in vitro* formation of microcalcifications has never been explored. We repeated our mineralisation experiment with a HER2 overexpressing sub-clone of the luminal MCF7 cell line^32^, to assess any difference in mineralisation capability between the parental and HER2 expressing cell lines. Similar to the parental cell line, no positive staining was detected in MCF7-HER2 cells in any treatment group (Fig. 1B).

We next examined the HER2 overexpressing SKBR3 and triple negative MDA-MB-231 cell lines. Both cells lines displayed positive Alizarin Red S staining by day 14 when cultured in OC + Dex media (Fig. 1C,D). Staining for both cell lines increased progressively over the 28-day time course, indicating a continual deposition of calcium. Positive staining was also observed in SKBR3 cells cultured in OC without dexamethasone, although the staining was significantly fainter and did not become apparent until day 28. Quantification of deposited calcium in SKBR3 cells cultured in OC + Dex media for 21 and 28 days showed a 3.5 (p < 0.05) and 4.2-fold (p < 0.01) increase relative to control values, respectively (Fig. 1E). MDA-MB-231 cells grown in OC + Dex media displayed a 9.4 (p < 0.01) and 17.1-fold (p < 0.0001) increase in deposited calcium at days 21 and 28 respectively (Fig. 1F).

### Alkaline phosphatase activity is essential for *in vitro* mineralisation

**Figure 2:**
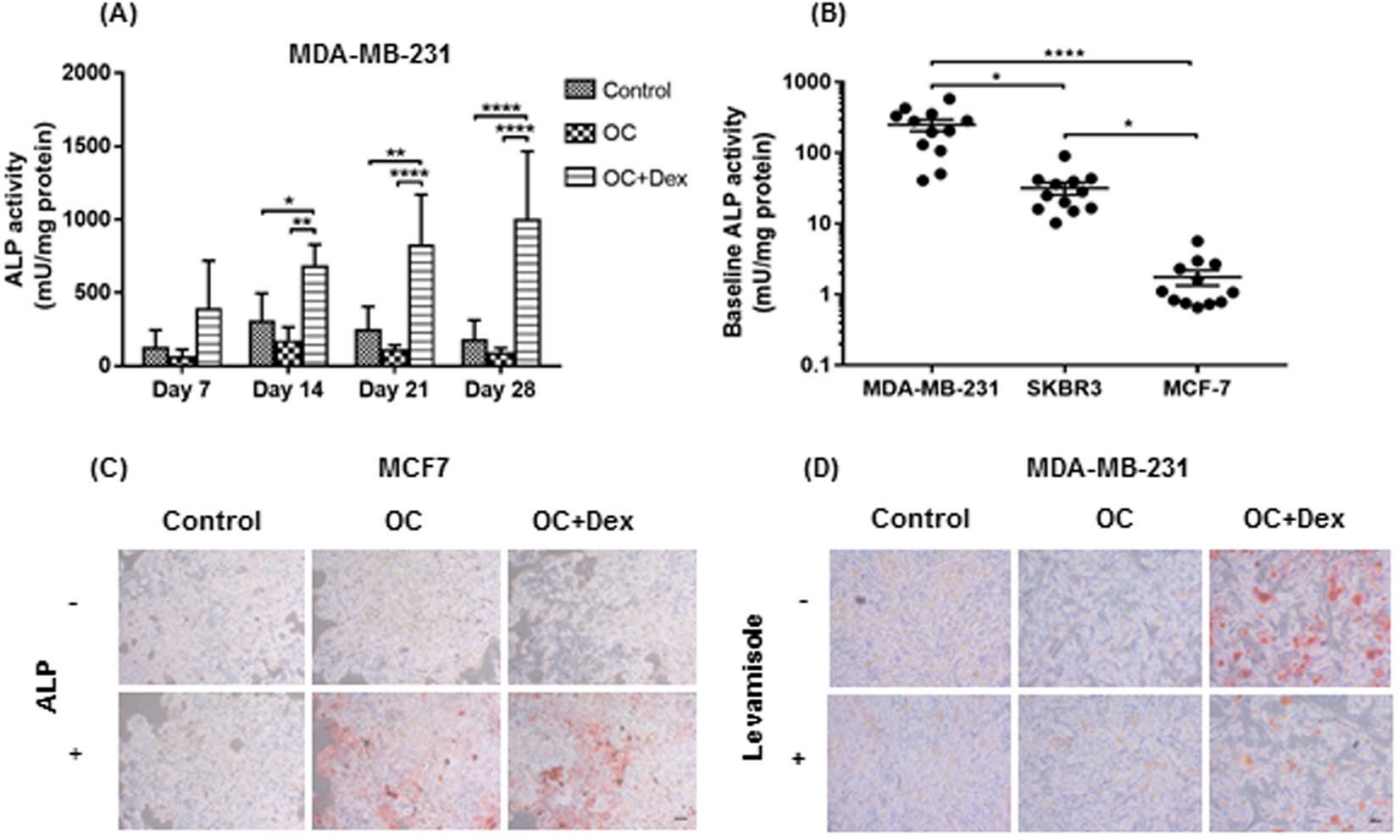
ALP activity as a key determinant of cellular mineralisation potential in human breast cancer cell lines. ALP activity was analysed in MDA-MB-231 cells (**A**) every 7 days by incubation with the ALP substrate p-nitrophenyl phosphate (PNPP), monitoring absorbance at 405 nm. ALP activity was normalised to protein content. Each point represents the mean + SD (n = 3), two-way ANOVA. *p < 0.05, **p < 0.01, ****p < 0.0001. ALP activity was compared between three cell lines (MDA-MB-231, SKBR3 & MCF7) by measuring “Control” values (3 replicates at 4 time points for a total of n = 12 for each cell line) (b). Alizarin Red S staining of MCF7 cells grown in control (regular growth media), OC (osteogenic cocktail) (50 µg/ml ascorbic acid and 10 mM β-glycerophosphate) or OC + Dex (OC + 100 nM dexamethasone) media supplemented with 1 U/mL exogenous bovine ALP or vehicle control (**C**), and MDA-MB-231 cells grown in control, OC or OC + Dex media supplemented with 100 µM levamisole or vehicle control (**D**). Representative images (n = 3) were captured at 10X magnification. Scale bar represents 50 µm.

Of central importance to our OC + Dex combination is the organic phosphate source β-glycerophosphate, which is degraded by the alkaline phosphatase (ALP) enzyme to release free phosphate (Pi), required for development of mineralisation^36^. Analysis of ALP activity in MDA-MB-231 cells did not reveal any significant differences in either control or OC treated cells throughout the 28 days, indicating that ALP activity in the MDA-MB-231 cell line remains relatively stable without additional stimulation (Fig. 2A). In contrast, the addition of dexamethasone promoted a strong induction of ALP activity, which increased continually throughout the time course, reaching a peak of 1004 mU/mg protein at day 28 (p < 0.0001). Levels of ALP activity were significantly greater in OC + Dex supplemented media compared to both control and OC groups, at days 14, 21 and 28.

A comparison of ALP activity in MDA-MB-231, SKBR3 and MCF7 cells grown in control media revealed significant differences in endogenous levels of ALP activity in each cell line (Fig. 2B). Even without the substantial increase of ALP activity induced by OC + Dex, MDA-MB-231 cells grown in standard DMEM media demonstrated a relatively high level of ALP activity, which was significantly elevated relative to ALP levels of both SKBR3 (p < 0.05) and MCF7 (p < 0.0001). Although levels of ALP activity in SKBR3 cells were lower than MDA-MB-231, they remained significantly elevated relative to the non-mineralising MCF7 cells line (31.98 mU/mg v 1.77 mU/mg, p < 0.05).

As the mineralisation capable MDA-MB-231 and SKBR3 cell lines displayed a significantly higher level of ALP activity than the non-mineralising MCF7 cell line, we hypothesised that increased ALP activity is a key difference between mineralising and non-mineralising cell lines. Addition of exogenous ALP promoted formation of mineralisation in MCF7 cells in both OC and OC + Dex media, as evidenced by the significant Alizarin Red S staining by day 7 (Fig. 2C). As observed previously, no positive staining was observed in OC or OC + Dex stimulated MCF7 cells in the absence of ALP, indicating no mineralisation. Finally, we found that addition of the ALP inhibitor levamisole significantly reduced Alizarin Red S staining in OC + Dex cultured MDA-MB-231 cells (Fig. 2D). Levamisole did not induce any difference in Alizarin Red S staining in either control or OC cultured cells.

### Expression of pro- and anti-mineralisation factors in MDA-MB-231 cells

*In vitro* formation of mineralisations is a complex process, occurring as the result of a carefully orchestrated programme, with contributions from a large number of effector proteins, regulatory factors, homeostatic pathways and feedback loops. Many the key players in physiological mineralisation are also found in close proximity to calcified deposits in a variety of pathological mineralisation diseases, suggesting a common pathway^37,38^.

**Figure 3:**
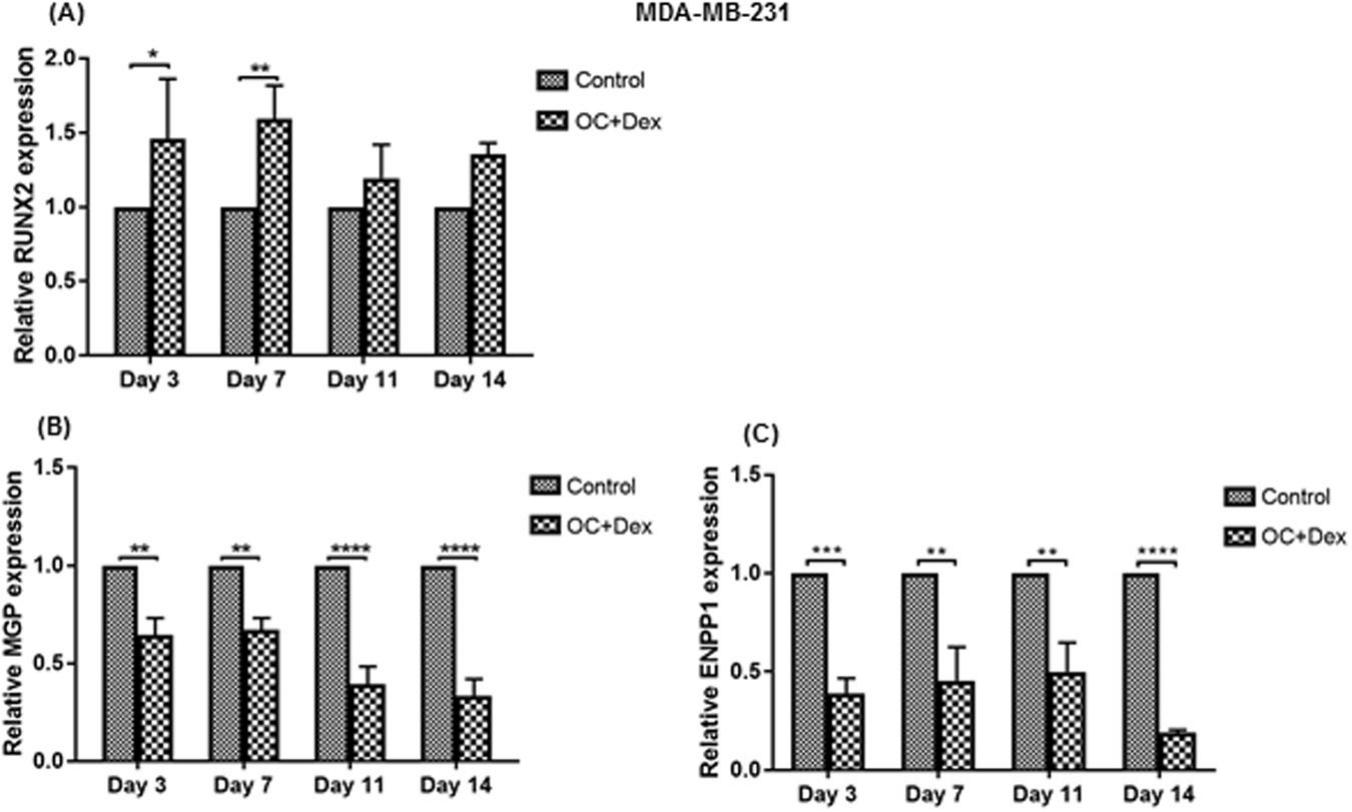
Altered expression of mineralisation regulating factors in MDA-MB-231 cells. Expression of RUNX2 (**A**), MGP (**B**) and ENPP1 (**C**) was analysed by qPCR in MDA-MB-231 cells cultured in control (regular growth media) or OC + Dex (50 µg/ml ascorbic acid, 10 mM β-glycerophosphate and 100 nM dexamethasone) media at the indicated time-points. Each time point represents the mean + SD (n = 3). qPCR results normalised to control values at each time point. Statistical significance was determined by two-way ANOVA, **p < 0.01, ***p < 0.001, ****p < 0.0001.

We observed 50% upregulation of the RUNX2 transcription factor at days 3 and 7 (Fig. 3A). RUNX2 is vital for generation of mature osteoblasts^39^, and is also upregulated in invasive breast tumours^40^. Conversely, when we examined expression of two key anti-mineralisation factors, we observed a time-dependent decrease. Expression of the vitamin-K dependent calcification inhibitor matrix gla protein (MGP) was significantly decreased in MDA-MB-231 cells cultured in OC + Dex media at all tested time points, with expression at day 14 decreased by approximately 70% (Fig. 3B, p < 0.0001). This decrease was also observed to be time-dependent, as levels at day 14 were significantly lower than days 3 and 7 (p < 0.01). The ectonucleotide pyrophosphatase/ phosphodiesterase 1 (ENPP1) enzyme produces the mineralisation inhibitor pyrophosphate through hydrolysis of extracellular ATP^41^, acting antagonistically to ALP. Treatment with OC + Dex strongly suppressed expression of ENPP1 by over 50% at all examined time points, with an 80% decrease observed by day 14 (Fig. 3C, p < 0.0001).

### Role of intracellular Ca^2+^ accumulation and TRPM7 channel activity in calcification formation

**Figure 4:**
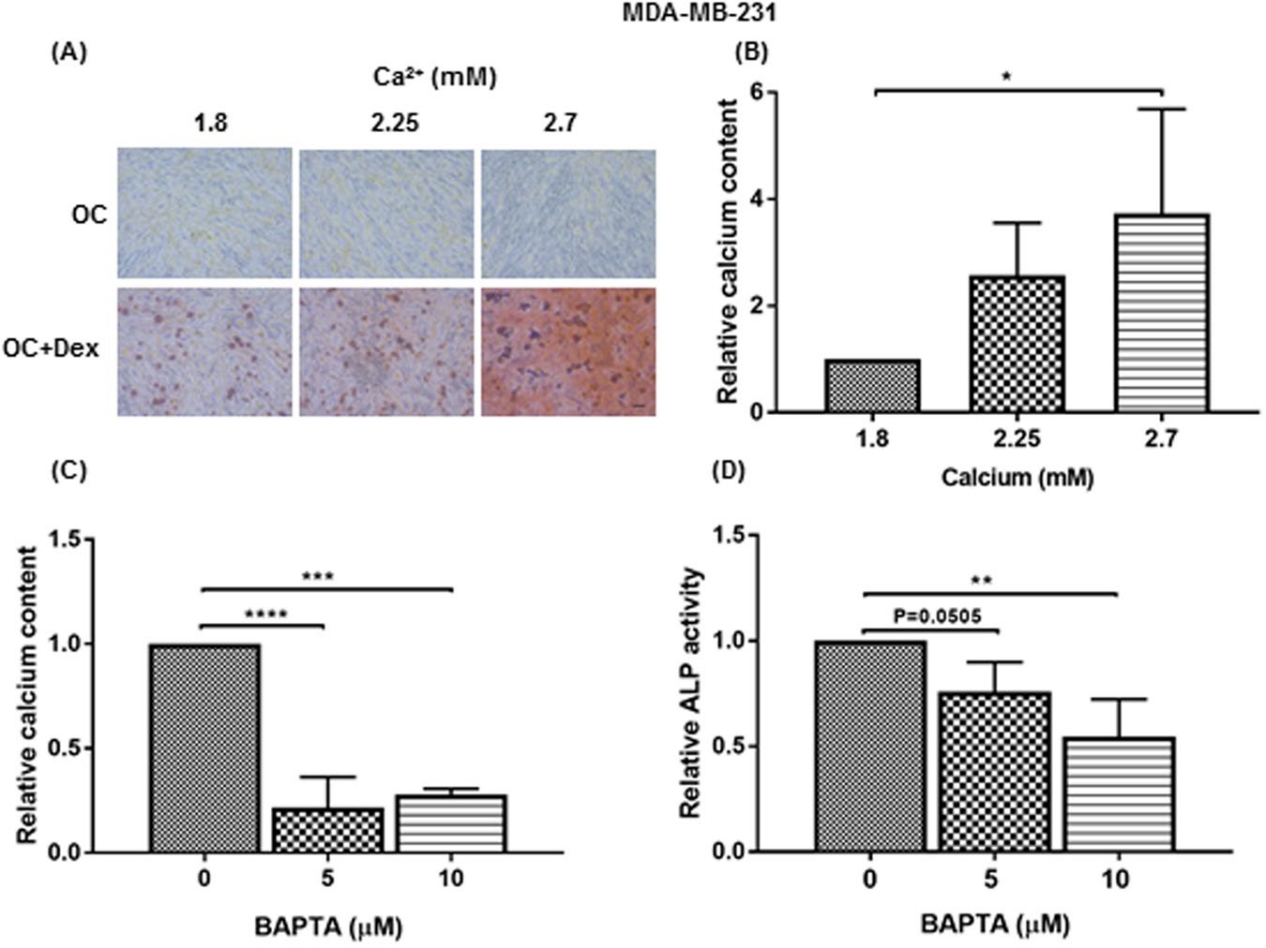
Relationship between extracellular and intracellular Ca^2+^ levels and development of MDA-MB-231 mineralisations. Representative images (10X magnification) of Alizarin Red S stained MDA-MB-231 cells in either OC (osteogenic cocktail) (50 µg/ml ascorbic acid and 10 mM β-glycerophosphate) or OC + Dex (OC + 100 nM dexamethasone) media at the indicated Ca^2+^ concentration (**A**). Scale bar represents 50 µm. Calcium content, as measured by OCP assay normalised to protein content revealed increased mineralisation in OC + Dex cultured cells at 2.7 mM Ca^2+^ (**B**). Each point represents mean + SD (n = 4). Usage of the intracellular Ca^2+^ chelator BAPTA-AM significantly decreased both calcification (**C**) and ALP activity (**D**). Each point represents mean + SD (n = 3). Statistical significance was determined by one-way ANOVA. *p < 0.05, **p < 0.01, ***p < 0.001, ****p < 0.0001.

Malignancy associated microcalcifications are composed of calcium phosphate crystals, most commonly hydroxyapatite^42^. Previously, we investigated the effect of ALP-induced Pi levels on the *in vitro* formation of calcifications. To assess if elevated Ca^2+^ levels may impact the development of calcifications, we supplemented DMEM media with calcium chloride to either 2.2 or 2.7 mM, representing a 25 and 50% increase in available Ca^2+^, respectively. Cells grown in OC media did not display any Alizarin Red S staining at either elevated Ca^2+^ levels, indicating that increased Ca^2+^ alone cannot promote mineralisation in our model in the absence of dexamethasone stimulation (Fig. 4A). In contrast, cells grown in OC + Dex media displayed increased Alizarin Red S staining when cultured at 2.2 or 2.7 mM Ca^2+^ compared to basal 1.8 mM. This was confirmed via quantification of extracted calcium deposits, with cells grown at 2.7 mM Ca^2+^ displaying an almost 4-fold increase in calcium measurements (Fig. 4B, p < 0.05). We next investigated if alterations in intracellular Ca^2+^ levels may be involved in the development of mineralisations. Chelation of intracellular Ca^2+^ was accomplished using BAPTA-AM, a membrane-permeable chelator complex that remains inactive until it crosses the cell membrane and has its acetoxymethyl ester group removed by intracellular esterases. 10 µM BAPTA-AM decreased MDA-MB-231 mineralisation by approximately 80% (Fig. 4C, p < 0.05). BAPTA-AM also reduced ALP activity of MDA-MB-231 cells by almost 50% (Fig. 4D, p < 0.01).

**Figure 5:**
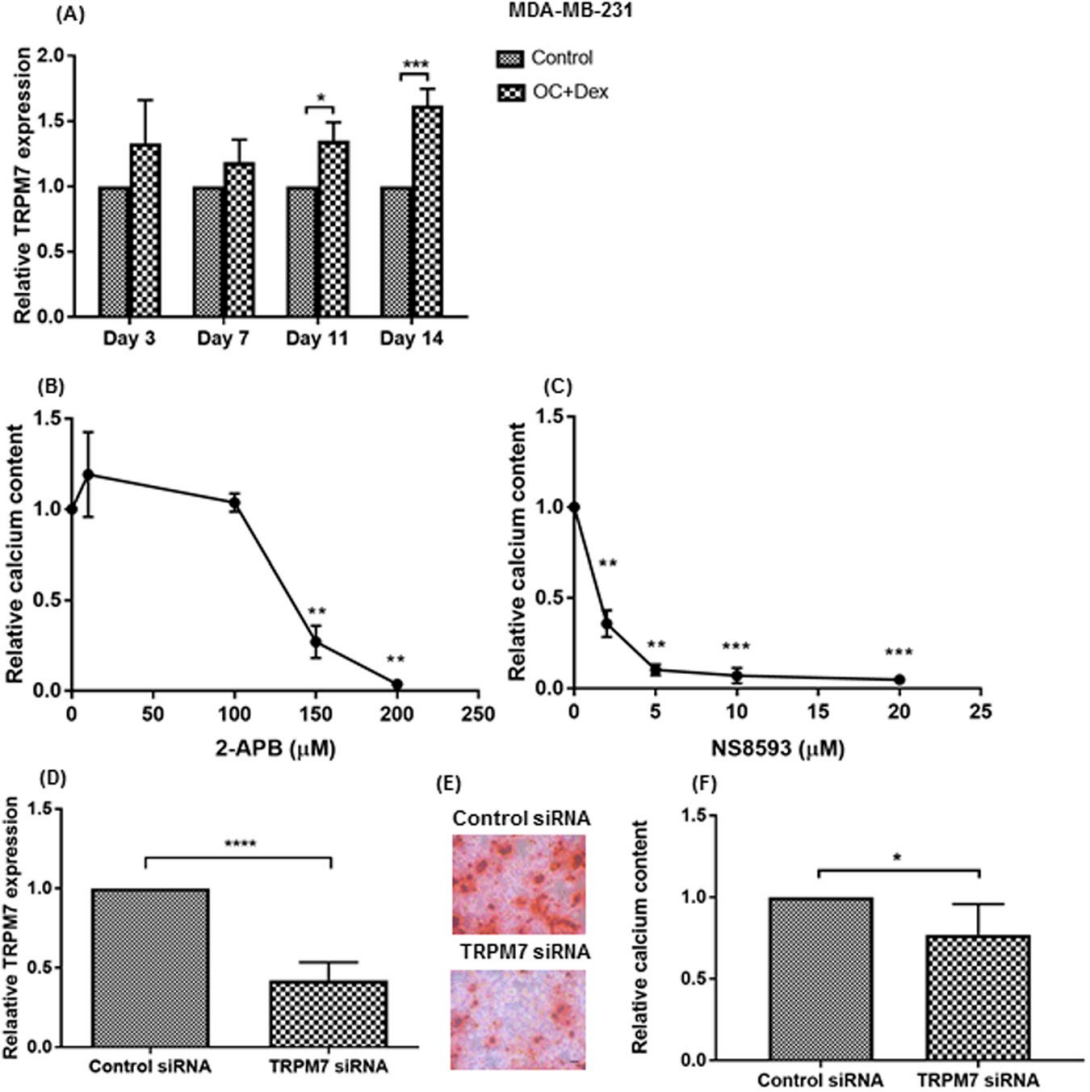
Role of TRPM7 channel in microcalcification formation. Expression of TRPM7 was analysed by qPCR in MDA-MB-231 cells cultured in control (regular growth media) or OC + Dex (50 µg/ml ascorbic acid, 10 mM β-glycerophosphate and 100 nM dexamethasone) at the indicated time-points (**A**). Each time point represents the mean + SD (n = 3). qPCR results normalised to control values at each time point. Calcium content of MDA-MB-231 cells cultured in OC + Dex media with 2-APB (**B**) or NS8593 (**C**) at the indicated concentration. Each value represents mean + SD (n = 3). ALP activity of MDA-MB-231 cells treated with 150 µM with 2-APB or 10 µM NS8593 (**D**). Decreased TRPM7 expression (**E**), Alizarin Red S (**F**) and calcium content (**G**) of MDA-MB-231 cells treated with TRPM7-targeting siRNA. Each point represents mean + SD (n = 5). Representative images shown at 10X magnification. Scale bar represents 50 µm. Statistical significance was determined by one-way ANOVA. *p < 0.05, **p < 0.01, ***p < 0.001, ****p < 0.0001.

These results suggest that an intracellular accumulation of Ca^2+^ is an important step in the development of microcalcifications, which concurs with previous studies^43^. The transient receptor potential melastatin type 7 (TRPM7) channel has been shown to be an important regulator of Ca^2+^ influx in breast cancer^44^ and has also been shown to be highly expressed in breast tumours with associated microcalcifications^45^. We found expression of TRPM7 to increase in response to OC + Dex media over a 14-day time course (Fig. 5A, 1.6-fold compared to control at day 14, p < 0.001).

To examine a potential role for the TRPM7 channel in the generation of microcalcifications, we tested the ability of MDA-MB-231 cells to mineralise under the effect of the TRP channel modulator 2-APB. Although no difference was observed for the two lowest tested concentrations (10 µM and 100 µM), the higher concentrations of 150 µM and 200 µM significantly decreased calcium, with 200 µM leading to a total inhibition of mineralisation (Fig. 5B, p < 0.05). To address any potential issues with the selectivity of 2-APB, we repeated the experiment using NS8593, a TRPM7 inhibitor with significantly improved specificity^46^. Results with NS8593 were even more significant, with all tested concentrations leading to a significant decrease in calcium content (Fig. 5C). NS8593 effectively blocked calcium deposition by over 65%, even in the lowest tested concentration of 2 µM, which roughly corresponds to the IC50 of the TRPM7 channel^46^. Finally, we also utilised siRNA to knockdown TRPM7 expression in MDA-MB-231 cells. Knockdown efficiency, as measured by qPCR at 72 hours, was 60% (Fig. 5D). Following a 72**-**hour incubation period with siRNA complexes, cells were switched to OC + Dex supplemented media for 14 days. Knockdown of TRPM7 significantly decreased both Alizarin Red S staining and calcium deposition (Fig. 5E,F).

### Breast calcifications and magnesium

**Figure 6:**
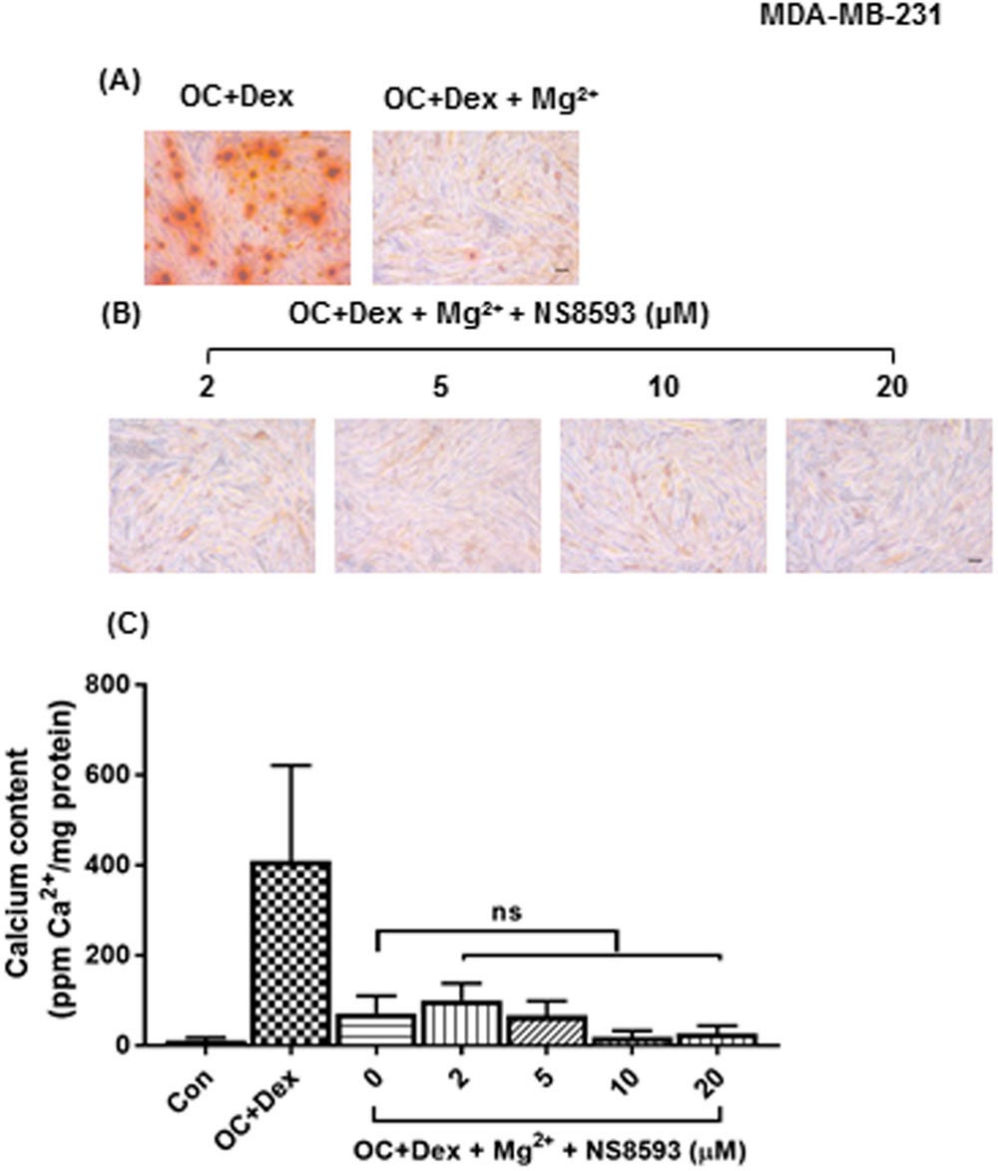
Anti-mineralisation effect of Mg^2+^. Representative Alizarin Red S staining of MDA-MB-231 cells cultured in OC + Dex media (50 µg/ml ascorbic acid, 10 mM β-glycerophosphate and 100 nM dexamethasone) with or without 0.7 mM Mg^2+^ supplementation (**A**). Alizarin Red S staining (**B**) and calcium content (**C**) of MDA-MB-231 cells cultured in OC + Dex media with 0.7 mM Mg^2+^ supplementation and NS8593 at the indicated concentration. Each point represents mean + SD (n = 3). Representative images shown at 10X magnification. Scale bar represents 50 µm. Statistical significance was determined by one-way ANOVA.

Interestingly, although we have shown evidence of a role for the TRPM7 channel in promoting calcification of breast cancer cells, several studies of other forms of pathological mineralisation have shown TRPM7 to exert a protective effect by promoting influx of Mg^2+^, a potent inhibitor of calcium deposition^47–49^. We first examined if Mg^2+^ had the same protective effect in our model as has been demonstrated in other studies. MDA-MB-231 cells were cultured under mineralising conditions in media with unaltered Mg^2+^ levels (0.8 mM) or DMEM supplemented with magnesium chloride to yield a total Mg^2+^ concentration of 1.5 mM, a concentration previously used in other studies of the anti-mineralisation effect of magnesium^49,50^. Increased magnesium concentration almost completely inhibited formation of microcalcifications (Fig. 6A). Addition of TRPM7 inhibitor NS8593 had no effect on the anti-mineralisation effect of Mg^2+^ (Fig. 6B,C) in contrast to other studies which have shown a partial reversal of Mg^2+’^s anti-mineralisation effect upon inhibition of TRPM7 channel activity^48,49^. Indeed, higher concentrations of the inhibitor seemed to further decrease calcification compared to Mg^2+^ alone, indicating that the protective effect of increased Mg^2+^ is independent of TRPM7 activity.

## Discussion

Despite their important role in the detection of breast cancer, the formation process of microcalcifications remains relatively understudied. As the majority of previous work in this area has utilised the murine 4T1 cell line^22,23^, we anticipated that expansion of the number of cell lines with characterised mineralisation potential would lead to novel findings. Similar to previous studies, we found the formation of mineralisations to be highly dependent on the properties of individual cell lines, with only triple-negative MDA-MB-231 and HER2-positive SKBR3 cell lines identified as mineralisation-capable, while neither parental nor HER2-overexpressing MCF7 cells displayed any positive staining (Fig. 1). Despite the known efficacy of microcalcifications in the clinical detection of luminal tumours^20^ it is worth noting the lack of calcification observed in the luminal MCF7 cell line. As with all *in vitro* models there may be limitations in the recapitulation of the complex interactions which may take place in an *in vivo* scenario. In addition, a recent study, Dang *et al*. observed positive mineralisation in MCF7 cells, in contrast to our own findings^24^. This discrepancy is explained by the modified version of our protocol utilised in their study, in which β-glycerophosphate was substituted with inorganic phosphate. Usage of inorganic phosphate results in an ALP-independent mechanism of mineralisation, as the phosphate necessary for calcium deposition is readily available to the cells without requiring ALP mediated degradation of β-glycerophosphate. We found similar results when we tested MCF7 cells with exogenous ALP (Fig. 2C).

The SKBR3 cell line represents the first identified HER2+ mineralising cell line, an important finding in light of the significant association between HER2 expression and the presence of microcalcifications^15,51^. Interestingly, we did not see positive results in HER2 overexpressing sub-clone of the luminal MCF7 cell line (Fig. 1B). Naseem *et al*. found microcalcifications in approximately 50% of HER2+ patients^20^. A similar figure of 56% was reported by Seo *et al*.^52^. These results indicate that although there is a strong tendency for HER2+ tumours to exhibit calcification, it is not a universal trait. It is likely that although HER2 may be contributing to the promotion of calcification, its expression is not sufficient by itself. In addition, HER2 expressing cell lines can differ significantly in their levels of HER2 expression, concomitant mutations and biological behaviour^53^, all of which may potentially alter their mineralisation capability. Future work to establish the exact contribution of HER2 expression to the formation of microcalcifications, including analysis of a larger panel of HER2 expressing cell lines would likely shed light on this question.

A vital feature of mineralisation-capable cell lines identified in this study was expression of the ALP enzyme, a key determinant of both physiological and pathological mineralisation^54^. Both MDA-MB-231 and SKBR3 cells displayed elevated levels of ALP activity relative to the non-mineralising MCF7 cell line (Fig. 2B) and in MDA-MB-231 cells, this was further enhanced by the presence of mineralisation promoting OC + Dex media (Fig. 2A). The importance of ALP was further established by diminished mineralisation of MDA-MB-231 cells when incubated with the ALP inhibitor levamisole (Fig. 2D) and findings of positive mineralisation of MCF7 cells following supplementation of culture media with exogenous ALP (Fig. 2C).

In models of both physiological and pathological mineralisation, ALP plays a two-fold role in the induction of mineralisation. Degradation of inhibitory pyrophosphate to free phosphate results in both a reduction in an anti-mineralisation agent and an increase in an important pro-mineralisation factor, tipping the balance in favour of calcium deposition. Pyrophosphate is produced primarily by the extracellular ENPP1 enzyme via hydrolysis of extracellular ATP^55^. When we examined expression of ENPP1, we observed a significant decrease over time in MDA-MB-231 cells following induction of mineralisation (Fig. 3C). Knockdown of ENPP1 expression in other models results in decreased pyrophosphate and extensive ectopic mineralisation^56^. Thus, the decreased ENPP1 expression we observed, in conjunction with the increased level of ALP activity could act in tandem by simultaneously decreasing the amount of pyrophosphate produced and increasing the enzymes necessary to degrade any pyrophosphate that is made.

In addition to ENPP1, we also observed altered expression of the mineralisation regulating genes RUNX2 and MGP (Fig. 3A,B). These findings indicate a shift in cellular behaviour towards a mineralisation-capable phenotype, in agreement with changes previously noted both *in vitro*^22,23^ and *in vivo*^28–31^. Both of these genes have also previously been found to play a role in breast cancer. RUNX2 has been shown to promote bone metastasis in breast cancer^57^ whilst MGP appears to act as a tumour suppressor in ER + breast cancer^58^, suggesting that the altered expression observed in these genes may help explain the unusually aggressive nature of calcification-associated tumours observed in some studies^10,59^.

In addition to changes in osteogenic expression, we also investigated the influence of tumour microenvironmental factors, in particular levels of Ca^2+^ and Mg^2+^ ions. Although a significant increase in calcium deposition was observed in cells grown in OC + Dex media with increased Ca^2+^, no positive mineralisation was seen in cells grown in media without Dex, indicating that elevated Ca^2+^ is insufficient by itself to induce mineralisation (Fig. 4A). Treatment of cells with BAPTA-AM to prevent intracellular Ca^2+^ accumulation significantly decreased both calcium deposition and ALP activity (Fig. 4C,D), indicating a requirement for Ca^2+^ uptake into the cell. These results, in conjunction with previous findings of diminished mineralisation in cells with impaired phosphate transport^22^ demonstrate a requirement for cellular uptake of both phosphate and calcium, and suggests an intracellular location for initial hydroxyapatite nucleation.

A review by Cross *et al*. proposed the formation of calcifications within the breast to involve alterations in Ca^2+^ transport pathways^26^. Although the findings of Dang *et al*., who demonstrated involvement of the secretory pathway Ca^2+^-ATPases transporters^24^, provided the first piece of *in vitro* evidence in support of this hypothesis, the majority of Ca^2+^ transporters remain uninvestigated. A previous study by Mandavilli found high immunohistochemical staining for the TRPM7 channel in breast tumours with associated calcifications^45^. TRPM7 expression in breast tumours has also been linked to proliferation^44^, epithelial–mesenchymal transition^60^ and tumour metastasis^61^. We found a time-dependent increase in TRPM7 expression following induction of mineralisation (Fig. 5A). Mineralisation of MDA-MB-231 cells was significantly inhibited following treatment with two different small-molecule inhibitors (Fig. 5B,C) and siRNA knockdown (Fig. 5E,F), strongly suggesting a functional role for the channel.

Interestingly, many of the previous studies on TRPM7 and calcification have shown it to exert a protective effect. TRPM7 is a non-selective cation channel, permeable to a range of ions and acts as an important regulator of Mg^2+^ homeostasis^62^. This role in Mg^2+^ influx seems to be essential to TRPM7 mediated protection^47–49^. In agreement with previous studies of other forms of pathological calcification, we found a potent anti-mineralisation effect of Mg^2+^ in our *in vitro* model, with an almost total suppression of calcium deposition in MDA-MB-231 cells cultured in 1.5 versus 0.8 mM Mg^2+^ (Fig. 6A). However, the anti-mineralisation effect of Mg^2+^ did not appear to be TRPM7 dependent in our model, as inhibition of TRPM7 channel did not reverse the suppression of mineralisation observed in MDA-MB-231 cells grown in OC + Dex media with increased Mg^2+^ levels (Fig. 6B,C). Although this finding is in disagreement with some of the literature, recently Zhang *et al*. demonstrated a pro-calcification effect for TRPM7 in vascular smooth muscle cells (VSMC)^63^. VSMCs grown in a calcifying media containing the same concentration of β-glycerophosphate used in our media formulation displayed a dose-dependent increase in calcification when stimulated with IL-18. This increase was completely blocked by either the TRPM7 inhibitor 2-APB or siRNA knockdown of TRPM7. In summary, although previous studies have tended to show an anti-mineralisation effect of TRPM7 in vascular calcification we have shown strong evidence of a pro-calcification effect for the channel in the context of breast microcalcifications.

**Figure 7:**
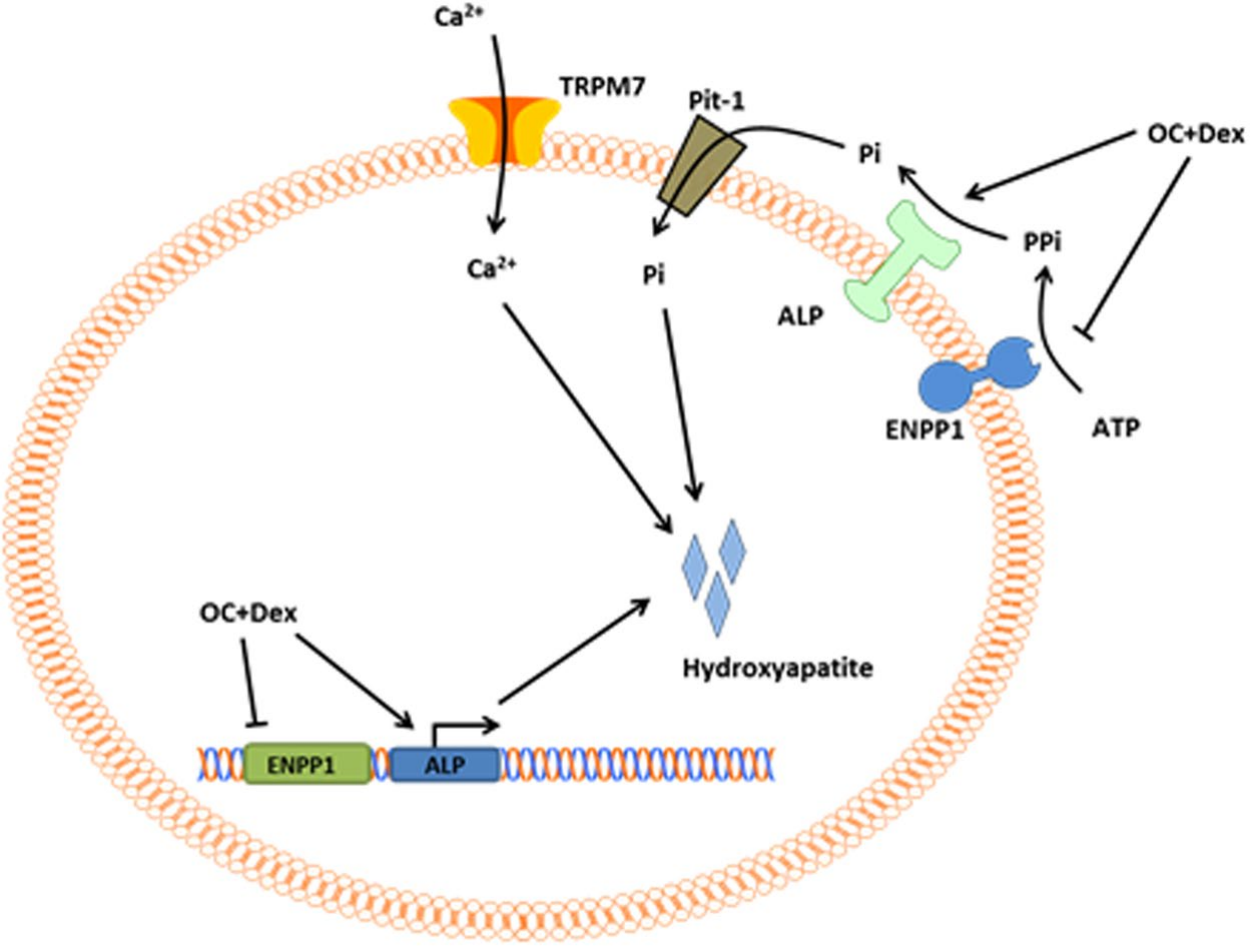
Proposed model of microcalcification formation. Extracellular nucleotide triphosphates (primarily ATP) are hydrolysed by activity of ENPP1 to release inhibitory pyrophosphate (PPi) which is further degraded by ALP. Free phosphate (Pi) enters the cell via the sodium-dependent phosphate transporter Pit-1^22^, where it can combine with Ca^2+^ entering, at least in part, via the TRPM7 channel to begin nucleation of hydroxyapatite. The OC + Dex media formulation used in our studies enhances several aspects of this process including upregulation of the pro-mineralisation ALP and RUNX2, and downregulation of the anti-mineralisation ENPP1.

Despite their central role in the clinical detection of a significant number of breast cancer cases every year, the formation of microcalcifications has not been extensively studied. We posit that the *in vivo* formation process of microcalcifications is a cellular-process involving phenotypic transformation of breast cancer cells to a mineralisation-capable state. This hypothesis is supported by the significant number of histopathological studies demonstrating altered expression of mineralisation associated proteins including BMP2, osteopontin, RUNX2, and TRPM7 in breast tumours with associated microcalcifications^28,29,31,45,64,65^. The model we are proposing requires activation of mineralisation-associated signalling pathways, influx of Ca^2+^ by transport proteins including TRPM7 and a cellular based formation of calcifications (Fig. 7). The data presented here, when combined with previous results from our group and others, has identified a significant number of mediators of calcification in an *in vitro* setting. Many of the genes we identified as potential contributors to formation of microcalcifications have previously been shown to influence breast tumour behaviour, potentially linking the formation of microcalcifications to increased tumour aggressiveness.

Although the direct translation of results obtained from cell lines to tumours remains a matter of debate, this model represents a starting point in an area, which has previously been underexplored. Work from both our group and others has also shown that xenograft models utilising human breast cancer cells can form *in vivo* mineralisations^22,66^, possibly indicating the next stage of research within this field that may allow for more physiologically relevant studies of the calcification process. This work builds on our previous findings and further demonstrates the role of a cell-mediated process in the formation of mammary calcifications.
